# Conservation Genetics of the Endangered Lompoc Yerba Santa (*Eriodictyon capitatum* Eastw., Namaceae), including Phylogenomic Insights into the Evolution of *Eriodictyon*

**DOI:** 10.3390/plants13010090

**Published:** 2023-12-27

**Authors:** C. Matt Guilliams, Kristen E. Hasenstab-Lehman

**Affiliations:** Santa Barbara Botanic Garden, Santa Barbara, CA 93105, USA; klehman@sbbotanicgarden.org

**Keywords:** *Eriodictyon*, Namaceae, ddRADseq, endangered, endemic, clonality

## Abstract

*Eriodictyon capitatum* (Namaceae) is a narrowly distributed shrub endemic to western Santa Barbara County, where it is known from only 10 extant California Natural Diversity Database element occurrences (EOs). Owing to low numbers of plants in nature, a limited overall extent, and multiple current threats, *E. capitatum* is listed as Endangered under the Federal Endangered Species Act and as Rare under the California Native Plant Protection Act. In the present study, high-throughput DNA sequence data were analyzed to investigate genetic diversity within and among all accessible EOs; to determine the extent of genetic isolation among EOs; to examine clonality within EOs; and to examine the taxonomic circumscriptions of *E. capitatum*, *E. altissimum*, *E. angustifolium*, and *E. californicum* through phylogenomic analysis. Population genetic analyses of *E. capitatum* reveal a pattern of strong genetic differentiation by location/EO. The clonality assessment shows that certain small EOs may support relatively few multilocus genotypes. The phylogenomic analyses strongly support the present-day taxonomic circumscriptions of both *E. altissimum* and *E. capitatum*, showing them to be reciprocally monophyletic and sister with strong support. Taken together, these results paint a picture of an evolutionarily and morphologically distinct species known from relatively few, genetically isolated stations.

## 1. Introduction

**Figure 1:**
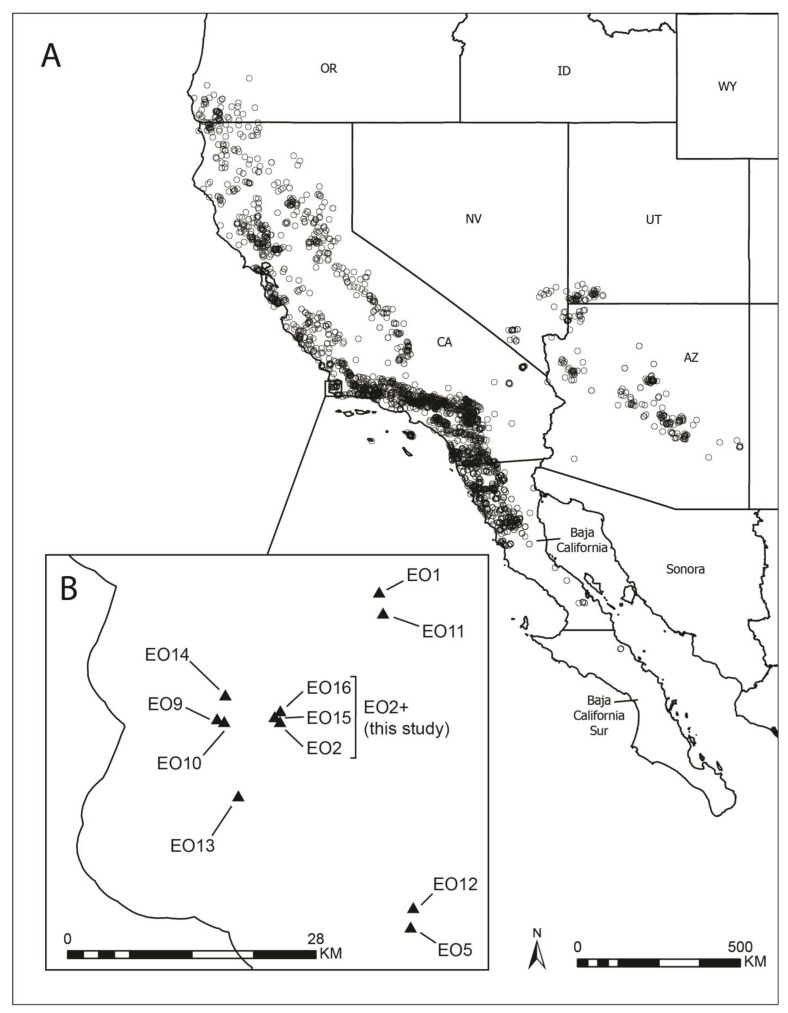
(**A**) Map of western North America showing distribution of *Eriodicyton* based on specimen records from the Global Biodiversity Information Facility (GBIF); (**B**) Inset map of Central California showing *E. capitatum* element occurrences (EOs) based on Kofron et al. (2022) [4].

*Eriodictyon* Benth. is a small genus of perennial herbs and shrubs endemic to western North America. It is typically delimited to include 11 species and 14 minimum-rank taxa, inclusive of species, subspecies, and varieties [1,2]. Based on phylogenetic evidence, *Nama rothrockii* A. Gray may be closely related to *Eriodictyon* as well, although no combination for this plant in *Eriodictyon* exists at the present time [1]. Members of the genus largely occur in the California Floristic Province [3], which extends from southwestern Oregon, United States, southward to northwestern Baja California, Mexico. The genus also has a second center of distribution in Arizona, Nevada, and Utah. Figure 1A shows the distribution of *Eriodictyon* based on specimen records available from the Global Biodiversity Information Facility (GBIF).

*Eriodictyon* is one of three genera in the Namaceae, a recently recognized family in the order Boraginales [5,6]. Recognition of this new family was motivated by previous molecular phylogenetic studies that revealed the Hydrophyllaceae s.l. to be non-monophyletic [1,7,8]. A family-level classification of the Boraginales and the rationale for recognizing Namaceae as distinct from Hydrophyllaceae can be found in Luebert et al. [5]. Currently recognized members of *Eriodictyon* are distinctive in the Namaceae as being rhizomatous shrubs, or in one case, a rhizomatous perennial herb [2]. Stems are usually erect and between one and four meters tall. Leaves are cauline, alternate, and linear, oblong, lanceolate, elliptical, or oblanceolate in shape. Inflorescences are usually borne at branch or twig apices and can be open or dense. Flowers have white to purple corollas that are usually either funnel- or urn-shaped. Plants are often glandular, usually with at least one plant organ producing an aromatic, sticky exudate. Fruits in *Eriodictyon* are typically small capsules with valvate dehiscence.

*Eriodictyon* species are considered to be short-lived, pioneer, or early successional taxa based on the observation that they often thrive in ecological settings that experience regular disturbance, such as fire-prone vegetation communities and roadsides [9,10,11]. In such settings, a single genetic individual may spread by rhizomes and produce a colony (genet) of one or more genetically identical stems (ramets). Following a disturbance that destroys some or all of the above-ground stems in a genet (e.g., fire, roadside clearing), new stems rapidly develop from existing rhizomes. Sexual reproduction by seed has been observed in some *Eriodictyon* taxa, with germination rates greatly enhanced by fire cues such as heat and charate [11,12,13]. Plants in the genus are reported to be obligately outcrossing; however, this is a life history feature that may result in minimal seed production in uniclonal stands [14].

**Figure 2:**
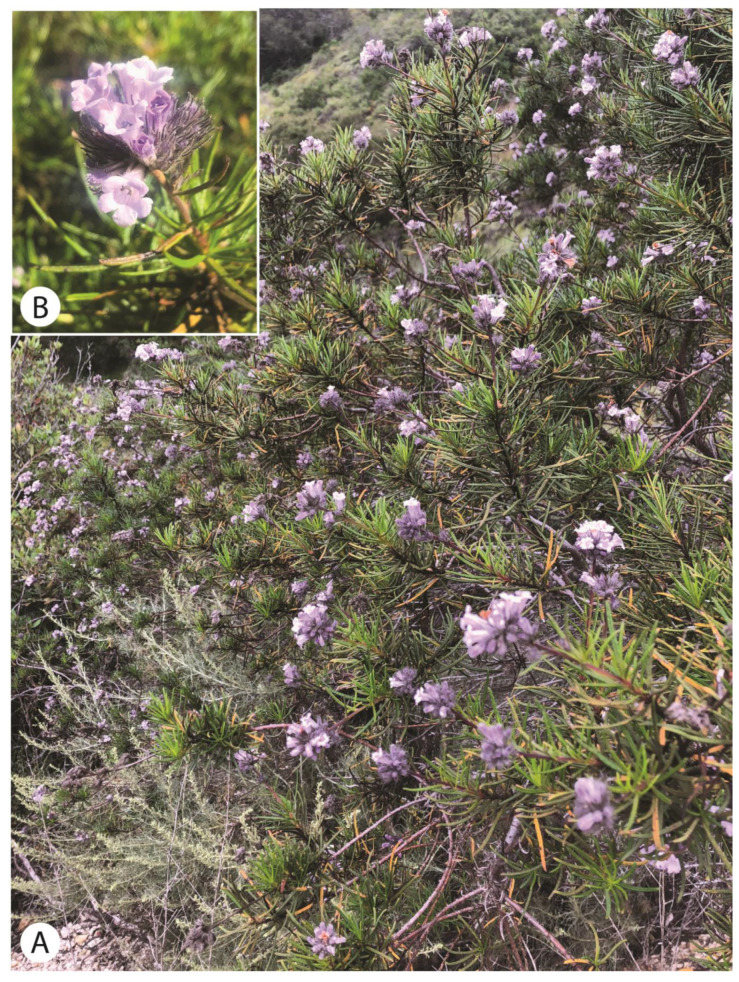
(**A**) Photograph of *Eriodictyon capitatum* in typical shrubland vegetation; (**B**) Head-like inflorescence of *E. capitatum*.

*Eriodictyon capitatum* Eastw. is a narrowly distributed shrub endemic to western Santa Barbara County (Figure 1B), where it grows in coastal settings in central coast maritime chaparral, bishop pine forest, and coastal scrub. Plants are usually less than three meters tall [2], but they have been observed to be up to five meters tall in some cases [4]. Leaves in *E. capitatum* are linear (Figure 2), an uncommon trait in the genus that it shares with only *E. altissimum* P.V. Wells of coastal San Luis Obispo County and *E. angustifolium* Nutt. of arid southeastern California, Nevada, Utah, and Arizona. *Eriodictyon angustifolium* also occurs disjunctly in northern Baja California, Mexico. As implied by the name, the inflorescences of *E. capitatum* are dense, head-like clusters of several flowers. The flowers have densely long-hairy calyces and lavender, funnel-form corollas. The combination of linear leaves, head-like inflorescences, and lavender, funnel-form corollas, is diagnostic for *E. capitatum* in the genus.

An evolutionary hypothesis involving *E. capitatum* was posited by Wells [9], who speculated that the geographically adjacent, Central California endemic *E. altissimum* may have arisen through historical hybridization between *E. capitatum* and the widespread *E. californicum* (Hook. & Arn.) Torr. He noted that *E. altissimum* has linear leaves morphologically similar to those of *E. capitatum* but open, glabrous inflorescences similar to those of *E. californicum*. He acknowledged that neither of these taxa occur in the vicinity of *E. altissimum* in the present day, an observation that would seem to weaken support for the hybrid origin hypothesis. To date, Wells’ idea has not been tested phylogenetically.

Owing to low numbers of plants in nature, a limited overall areal extent, and multiple current threats, *E. capitatum* is listed as Endangered under the Federal Endangered Species Act, as Rare under the California Native Plant Protection Act, and has been given the California Rare Plant Rank of 1B.2 by the California Native Plant Society. As a rare plant, the taxon is tracked by the State of California in the California Natural Diversity Database (CNDDB). *Eriodictyon capitatum* is known from only 10 extant CNDDB element occurrences (EOs). An EO is defined as a specific location where a taxon of conservation concern has been documented as occurring. By convention, observations of individuals and/or populations of focal taxa are grouped together into one EO when the distance between them is less than ¼ mile. As a result, EOs may be composed of several or even dozens of biological populations or be limited to one or only a few individuals. Six of the extant *E. capitatum* EOs are on Vandenberg Space Force Base, and four are on private property. See Kofron et al. [4] for detailed information about each *E. capitatum* EO.

Elam [14] studied several aspects of *E. capitatum* in six populations as part of her doctoral research. Note that these six populations are now treated as belonging to three present-day EOs. She used starch gel electrophoresis of isozymes to examine clonality, inferring a wide range of clonality levels among the populations based upon gel banding patterns. In two populations, all sampled ramets had identical banding patterns and were assumed to belong to the same genet. In other populations, the numbers of inferred genets were much greater than one. A total of 17 unique isozyme banding patterns were detected among 26 sampled ramets in one population on Hollister Ranch (65 percent unique patterns), each assumed to represent a distinct genet. Seed production per sampled ramet was assessed by direct counts over two years. Seed production varied significantly between populations in both years, with population 3 on Vandenberg Space Force Base producing considerably more seed (40.3 in 1992 and 72.6 in 1993) than the other sampled populations (0.4–2.3 in 1992 and 0.5–9.1 in 1993). Self-incompatibility was assessed within multi-clonal populations by hand self- and cross-pollinations between ramets, as determined by earlier isozyme banding patterns. Inflorescences were bagged after hand pollination. Mean fruit and mean seed production for each ramet were quantified. The percent of flowers setting seed was significantly higher (t = 5.18, *p* < 0.001) in hand cross-pollinated inflorescences (mean = 53.1) than hand self-pollinated inflorescences (mean = 1.9). Seeds per fruit were also significantly different between treatments (t = 4.47, *p* < 0.002), with a mean of 1.77 seeds per fruit for hand cross-pollinations and only 0.03 seeds per fruit for hand self-pollinations. Finally, the relationship between mean seed production and clonal diversity per population was assessed, but statistical tests were either not significant or only marginally significant in the two study years.

Although the plant’s rarity and listing status have resulted in considerable conservation focus, much remains to be learned about *E. capitatum*. While Elam’s work provided critical insight into several aspects of the species’ biology, it focused on only three present-day EOs and used an older approach to assess genetic diversity. Therefore, it would be useful to examine the magnitude of genetic diversity in present-day EOs using an updated approach. It would also be useful to better understand the extent to which EOs are genetically distinct from one another. Although Elam examined clonality in certain *E. capitatum* EOs, clonality has not been assessed using DNA sequence data. Finally, the phylogenetic and taxonomic distinctiveness of *E. capitatum* with respect to putative close relatives *E. altissimum*, *E. angustifolium*, and *E. californicum* has never been evaluated using molecular tools (but see Vasile et al. [15] for a recent phylogenetic analysis that included some members of *Eriodictyon*). Doing so would permit the evaluation of Wells’ *E. altissimum* hybrid origin hypothesis. Leveraging the utility of high-throughput sequencing to resolve some or all of these data gaps would be useful in the case of *E. capitatum*, allowing resource agencies and land managers to use this information for conservation planning.

Here we generated a SNP dataset using high-throughput sequencing and used it to (1) investigate the genetic diversity within and among all accessible *E. capitatum* EOs; (2) determine the extent of genetic isolation among EOs of *E. capitatum*; (3) examine clonality within EOs of *E. capitatum* (i.e., how many unique genets are there within sampled ramets of an element occurrence); and (4) use phylogenomic analysis to evaluate the evidence for the current taxonomic circumscriptions of *E. capitatum*, *E. altissimum*, *E. angustifolium*, and *E. californicum,* thereby assessing Wells’ *E. altissimum* hybrid origin hypothesis.

## 2. Results

### 2.1. Population Genomic Analyses

Dataset 1 included 85 samples with 200,186 SNPs and an aligned matrix of 2,778,920 bps. The percent of missing data was 31.07% in the SNP matrix. Summary statistics by sample are provided in Supplementary Table S1.

Table 1 provides population genomic summary statistics for each EO averaged across all loci, including: number of individuals from each population/EO (*N*), mean individuals genotyped at each locus (*n*), number of private alleles (Private), mean frequency of the major allele (P), observed heterozygosity (*H*o), expected heterozygosity (*H*e), nucleotide diversity (Pi), and the mean Wright’s inbreeding coefficient (*F*_IS_). The number of individuals sequenced per EO (*N*) ranged from 12 to 20. The average number of individuals genotyped at each locus (*n*) ranged from about 7 to 15.7. Private alleles per EO ranged from 8572 to 32,246. The mean frequency of the major allele (P) was quite high, ranging from 0.917 to 0.977. Observed heterozygosity (*H*o) was lower than expected heterozygosity (*H*e) in three EOs (EO1, EO2+, and EO5) and greater or about equal to expected heterozygosity in EO9, EO13, and EO14. Nucleotide diversity (Pi) ranged from 0.045 in the small La Salle population (EO13) to 0.127 in the largest EO, Orcutt Hill (EO1). Mean Wright’s inbreeding coefficient (*F*_IS_) ranged from −0.009 to 0.205. Calculated *F*_ST_ values are provided in Table 2. Values range from 0.114 between EO1 and EO2 to 0.417 between EO13 and EO14.

**Figure 3:**
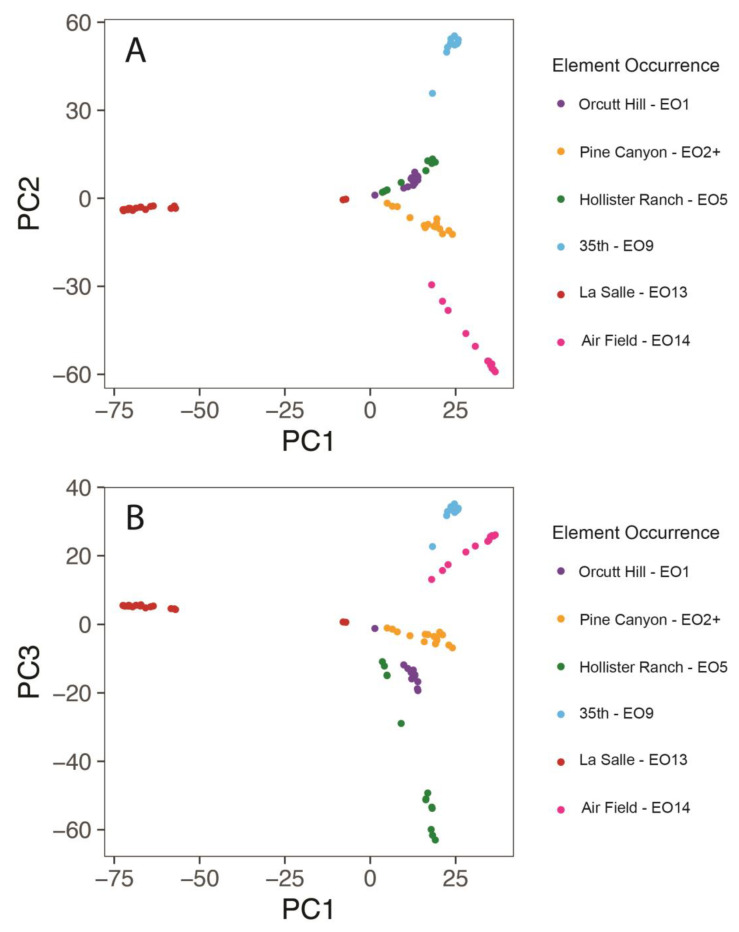
Scatter plots of (**A**) PC2 vs. PC1 and (**B**) PC3 vs. PC1.

Principal components (PCs) 1, 2, and 3 explained 18.6, 11.2, and 8.2 percent of the variability in the PCoA analysis, respectively. Scatterplots of PC2 versus PC1 and PC3 versus PC1 are given in Figure 3A,B. In nearly all cases, samples cluster tightly by EO, and clusters of samples by EO are usually not overlapping with other samples. There are exceptions in both cases. In Figure 3A,B, there are two samples from La Salle EO13 that do not cluster with the other samples from this EO. In Figure 3B, there is one sample from Orcutt Hill EO1 that appears near Pine Canyon EO2. In Figure 3A, the samples from Orcutt Hill EO1 and Hollister Ranch EO5 overlap strongly but form non-overlapping clusters in Figure 3B.

**Figure 4:**
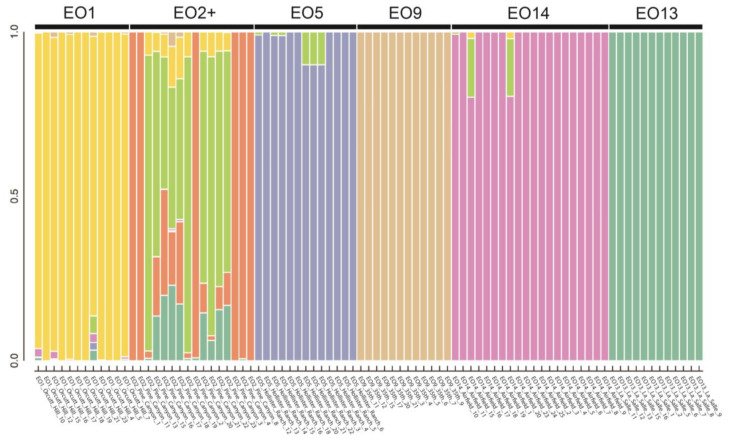
STRUCTURE barplot for K = 7, averaged across replicates. Inferred genetic subdivisions are each represented by a color.

Table 3 shows the summary statistics associated with the STRUCTURE analysis. The highest deltaK value was associated with genetic subdivisions (K) = 7 (557.149). Figure 4 shows the STRUCTURE barplot for K = 7, averaged across replicates. Inferred genetic subdivisions are each represented by a color. Vertical bars represent individual samples, which are labeled along the *x*-axis. Samples are grouped by EO. Five out of six EOs contain samples that are assigned entirely to the same genetic subdivision. Only Pine Canyon EO2 has samples assigned to multiple genetic subdivisions. Two of these genetic subdivisions are unique to Pine Canyon EO2, suggesting a likely genetic substructure within this EO.

**Figure 5:**
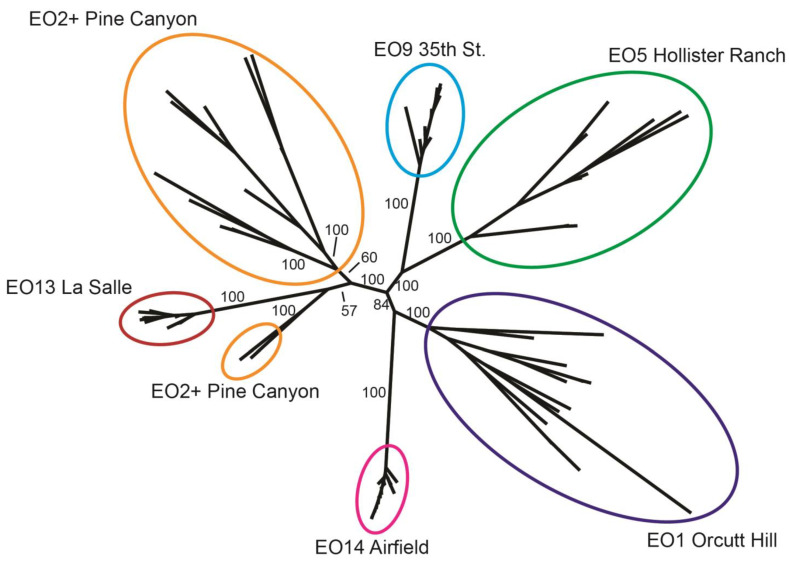
Unrooted phylogenetic tree inferred using maximum likelihood in RAxML. Internal nodes are annotated with maximum likelihood bootstrap support values. Colored ellipses denote sampled element occurrences.

**Figure 6:**
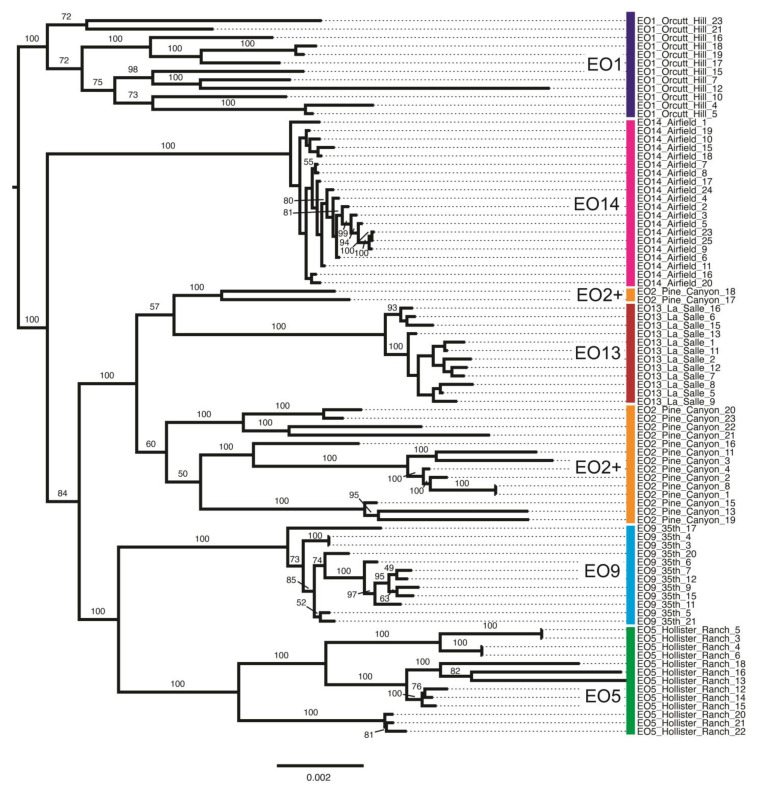
Phylogenetic tree inferred using maximum likelihood in RAxML, arbitrarily rooted on the EO1 clade of samples. Nodes are annotated with maximum likelihood bootstrap support values above 50.

The tree diagrams resulting from the phylogenetic analysis of Dataset 1 using ML in RAxML are shown in Figure 5 and Figure 6. Figure 5 shows an unrooted tree without sample names or the majority of bootstrap support values, so that overall patterns by EO are more apparent. Groupings of samples by EO are indicated with colored ellipses. In nearly all cases, deep relationships in the tree are strongly supported (e.g., ML bootstrap = 100). Samples form well-supported clades by EO in all cases but Pine Canyon. For Pine Canyon samples, most form a single large grouping with poor support (ML bootstrap = 60), sister to a grouping of EO13 samples + two additional Pine Canyon samples. This latter grouping of EO13 + two Pine Canyon samples is also poorly supported (ML bootstrap = 57). For this reason, the placement of these two Pine Canyon samples is somewhat equivocal.

Figure 6 shows the same phylogenetic tree as Figure 5, but rooted arbitrarily on the EO1 clade. In this figure, all sample/tip labels are shown, as are the ML bootstrap values for each branch. Samples by EO are denoted by labeled vertical bars. Note that grouping patterns are identical between Figure 5 and Figure 6, having been based on the same ML tree. Relationships and statistical support within each EO are more readily apparent, however, as are branch lengths. For example, note relatively long branch lengths within EO1 and EO2, and relatively short branch lengths within EO14 and EO9.

### 2.2. Clonality

**Figure 7:**
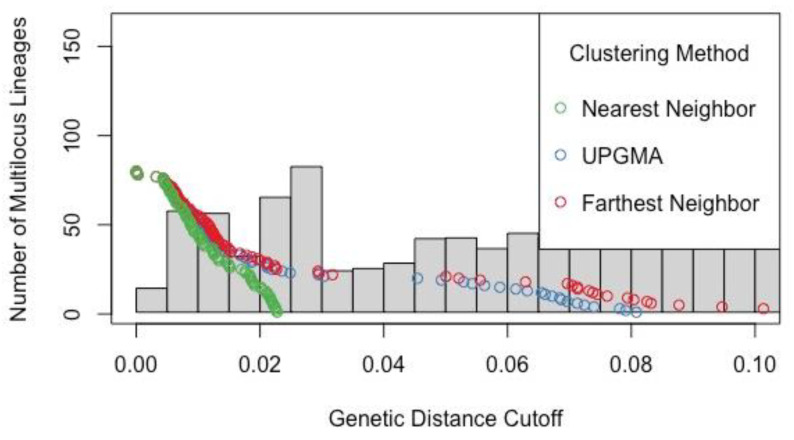
Filter_stats plot from R package poppr showing the number of multilocus lineages that result from different genetic distance cutoff values under three different clustering methods: nearest neighbor (green circles), UPGMA (blue circles), and farthest neighbor (red circles). A histogram of all pairwise genetic distances is shown as gray bars.

The filter_stats function of the R package poppr v2 [16,17] was used to produce the plot in Figure 7. This plot shows a histogram of all pairwise genetic distances between samples (gray bars) along with the number of multilocus genotypes (called “multilocus lineages” on the *y*-axis) that would result for all genetic distance cutoff values (*x*-axis) under the three clustering methods. A large gap in the inferred multilocus genotypes under the farthest neighbor method (red circles) is evident between genetic distance cutoff values of approximately 0.0225 and 0.025.

The cutoff.predictor function in the R package poppr was used as one approach for identifying a threshold genetic distance at which individual samples would be assigned to multilocus genotypes under the conservative farthest neighbor clustering method. This function returned a threshold of 0.001747, which was much lower than the evident gap in inferred multilocus genotypes under the farthest neighbor method (red circles).

Multilocus genotype assignments were made using the mlg.filter function in the R program poppr v2 using two thresholds. First, the threshold resulting from the cutoff.predictor function of 0.001747 was used, despite appearing illogically low. Using this threshold, the 81 samples that passed filtering steps were assigned to 78 total multilocus genotypes. Because this result is essentially uninformative and likely based upon a spurious and arbitrarily low threshold, the results following the use of this threshold are not discussed further. Second, a threshold corresponding to the gap inferred in the multilocus genotypes of 0.025 was used (see Figure 7). Using this threshold, the 81 samples that passed filtering steps were assigned to 25 total multilocus genotypes.

The number of multilocus genotypes inferred under the 0.025 genetic similarity threshold differed among EOs. Table 4 lists the number of multilocus genotypes inferred by EO. Supplementary Table S2 provides the multilocus genotype assignment for each sample. In no case were multilocus genotypes shared between or among EOs.

### 2.3. Phylogenomics

**Figure 8:**
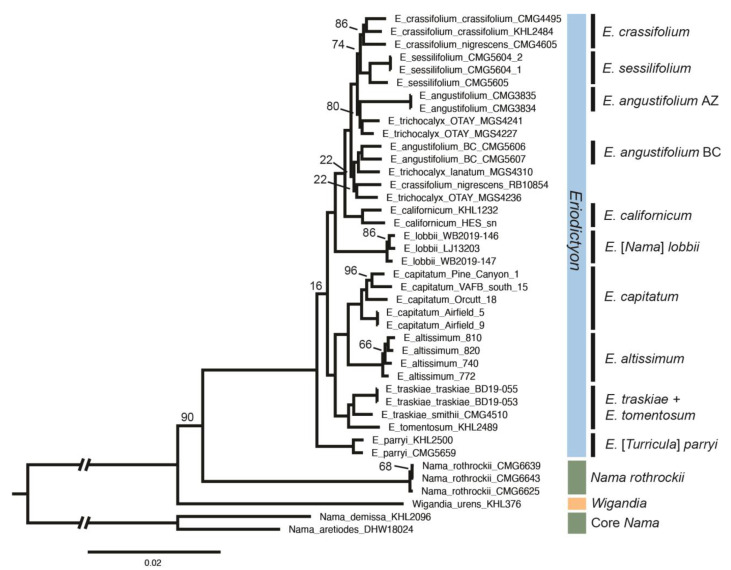
Phylogeny of *Eriodictyon* inferred using maximum likelihood in RAxML. Maximum likelihood support values are 100, except as noted.

The full Phylip dataset constructed in ipyrad for the purpose of inferring phylogenetic relationships contained 41 samples and was 2,248,102 base pairs long. The ML phylogenetic tree resulting from the RAxML analysis is shown in Figure 8. In general, the tree topology is well supported, with most clades supported with bootstrap values of 100. Bootstrap values less than 100 are indicated on the tree. Only five branches have bootstrap values below 70. Two of these low values occur at shallow phylogenetic depths of divergence among samples of the same taxon (e.g., 68 within a clade of *Nama rothrockii* samples). The other three low ML bootstrap values occur at deeper depths of divergence. Of note is a low bootstrap value (ML bootstrap = 16) near the base of *Eriodictyon*, which, given the surrounding strong pattern of statistical support, likely indicates multiple +- equally likely placements for samples of *E*. [*Turricula*] *parryi* (A. Gray) Green.

The *Eriodictyon* clade has the highest possible statistical support (ML bootstrap = 100). Most *Eriodictyon* samples in the analysis form strongly supported clades by taxon. This includes *E. altissimum*, *E. californicum*, *E. capitatum*, *E. crassifolium* Benth. (all but one sample), *E*. [*Nama*] *lobbii*, *E*. [*Turricula*] *parryi*, *E. sessilifolium* Greene, and *E. traskiae* Eastw. Of these clades of samples by taxon, only the *E. crassifolium* clade is supported by a bootstrap value of less than 100 (ML bootstrap = 86).

Of the *Eriodictyon* taxa in this analysis, only the *E. angustifolium* and *E. trichocalyx* samples do not form clades by taxon. *Eriodictyon angustifolium* samples form two strongly supported clades by region: Arizona and Baja California. These *E. angustifolium* clades by region are not closely related in this analysis. Similarly, samples of *E. trichocalyx* appear in two different parts of the tree, although support is weak in one of these regions.

Phylogenetic relationships among *E. altissimum*, *E. angustifolium*, *E. californicum*, and *E. capitatum* are relatively well resolved in this analysis, and support for current taxonomic circumscriptions is robust for all but *E. angustifolium*. Both narrow-leaved Central Coast taxa, *E. altissimum* and *E. capitatum*, have samples that form well-supported clades (each with ML bootstrap = 100). The *E. altissimum* clade is sister to the *E. capitatum* clade with strong support (ML bootstrap = 100), and this is sister to a well-supported clade of central coast and southern California taxa. The two included samples of *E. californicum* form a well-supported clade (ML bootstrap = 100) that is relatively distantly related to *E. altissimum* and *E. capitatum*. Although narrow-leaved *E. angustifolium* samples do not form a single clade, both groupings of samples of *E. angustifolium* are relatively distantly related to *E. altissimum* and *E. capitatum*.

Outgroup sampling for this project permitted the evaluation of the placement of *E. lobbii*, previously included in the genus *Nama*, *E. parryi*, previously included in *Turricula*, and *Nama rothrockii*. Samples of *E*. [*Nama*] *lobbii* form a clade with strong support (ML bootstrap = 100) that is resolved within *Eriodictyon*. The two included samples of *E*. [*Turricula*] *parryi* form a clade with strong support (ML bootstrap = 100) that is placed sister to the remainder of *Eriodictyon* in the best ML tree, but with poor statistical support; the *E. parryi* clade is sometimes placed within *Eriodictyon* in bootstrap replicates. Samples of *Nama rothrockii* also form a clade with strong support (ML bootstrap = 100); this was recovered on a relatively long branch sister to a strongly supported (ML bootstrap = 100) clade of *Eriodictyon* samples.

## 3. Discussion

### 3.1. Population Genomic Analyses

Genetic diversity among the sampled EOs is variable, but the overall patterns are consistent with EO census sizes and varying areal extents. The EO with the highest nucleotide diversity (0.127) and second largest number of private alleles (31,142) is Orcutt Hill (EO1), which has the second highest census count (>7000 ramets in 2018) and a large areal extent. Similarly, with the second highest nucleotide diversity (0.123) and largest number of private alleles (32,246), the Pine Canyon EO (EO2+) also supports the largest number of ramets (9794 stems in part in 2015) over a relatively large area. In contrast, the Air Field location (EO14) has the lowest nucleotide diversity (0.031) and the second-smallest number of private alleles (14,296), which corresponds with a low census count (78 ramets in 2018) and small areal extent. Similarly, the newly discovered La Salle location (EO13) has the second lowest nucleotide diversity (0.045) and the lowest number of private alleles (8572), a finding consistent with the low ramet count (258 ramets in 2018) and small areal extent.

Despite proportionally higher genetic diversity in the larger EOs, these locations also had higher inferred levels of inbreeding, as suggested by lower observed heterozygosity estimates relative to expected heterozygosity (e.g., 0.059 observed and 0.118 expected in EO1) and higher mean Wright’s inbreeding coefficients (e.g., 0.186 and 0.205 for EO1 and EO2, respectively). This suggests that despite having a large number of above-ground *E. capitatum* stems at these locations, some factor (e.g., clonality) might be affecting estimates of heterozygosity and inbreeding.

Interpretation of *F*_ST_ values is straight-forward in some cases but not in others. The lowest *F*_ST_ value (0.114) was between Orcutt Hill (EO1) and Pine Canyon (EO2+). Although not as close together as some pairs of EOs, Pine Canyon is the closest sampled EO to Orcutt Hill. In contrast, Hollister Ranch (EO5) has a relatively low *F*_ST_ with respect to both Orcutt Hill (EO1) and Pine Canyon (EO2+), despite relatively large distances between EOs in both cases (*F*_ST_ 0.170 and 0.163, respectively). The largest *F*_ST_ values, suggesting the largest pairwise degree of genetic differentiation, involve EOs 9, 13, and 14, in most cases, which are the western-most EOs of *E. capitatum*. The *F*_ST_ value for the EO9–EO13 comparison was 0.345; for the EO9–EO14 comparison, the value was 0.357; and for the EO13–EO14 comparison, the value was 0.417. This finding is unexpected given the relative geographic proximity of EO9, EO13, and EO14.

Visualizing genetic distances using a PCoA ordination approach revealed strong genetic similarities among samples within EOs. It also showed intriguing patterns among EOs. In the scatterplot of PC2 versus PC1 (Figure 3A), the clusters of samples from 35th EO9, La Salle EO13, and Air Field EO14 were mostly well-separated from all other EOs, suggesting minimal gene flow between each of these and all other EOs. The other three EOs—Orcutt Hill EO1, Pine Canyon EO2+, and Hollister Ranch EO5—were relatively tightly clustered together in ordination space. This finding is surprising for Hollister Ranch EO5, given its relative geographic distance from Orcutt Hill EO1 and Pine Canyon EO2+. This finding is consistent with the calculated *F*_ST_ values, however. In the scatterplot of PC3 versus PC1 (Figure 3B), the La Salle EO13 was again strongly separated from all other EOs, but 35th EO9 and Air Field EO14 were close together in the plot. As in the first scatterplot, Orcutt Hill EO1, Pine Canyon EO2+, and Hollister Ranch EO5 were relatively close together in the plot.

The STRUCTURE results found genetic subdivisions in the data that corresponded almost perfectly with location (EO). Only Pine Canyon EO2+ had samples assigned primarily to more than one genetic subdivision. In this EO, some samples were assigned to one EO-specific subdivision colored orange, while the other samples were assigned primarily to an EO-specific subdivision colored bright green and, to a lesser extent, orange. These latter samples were also assigned with less likelihood to genetic subdivisions found in other EOs, including Orcutt Hill EO1 and La Salle EO13. These results strongly support the genetic distinctiveness of each *E. capitatum* EO and suggest a potential genetic substructure in Pine Canyon EO2+.

The patterns in the RAxML phylogenetic trees applied to Dataset 1 reinforce the findings of earlier analyses. Basic population genetic summary statistics revealed that each EO contained moderate and sometimes unique genetic diversity (based on nucleotide diversity and private alleles), and pairwise *F*_ST_ values showed moderate genetic divergence in all combinations. These results are supported by the inference of robustly supported clades of *E. capitatum* samples by EO and long branch lengths in some tips of the tree (samples). Beyond corroborating other findings, the tree diagram presented in Figure 7 may be useful in the future for selecting genetically dissimilar stems within an EO, e.g., for use in hand-crossing experiments where an emphasis would be on crossing stems/ramets that belong to different genets, as was conducted by Elam [14].

### 3.2. Clonality

Examination of clonality using high-throughput sequence data, which by its nature may include non-trivial amounts of missing data, requires careful assessment. Assigning samples to the same genetic individual based on genetic identity between or among samples is not practicable given certain properties of the data that result from certain high-throughput sequencing approaches, such as ddRADseq. Kamvar et al. [16,17] recommend a thresholding approach in which samples are collapsed to multilocus genotypes when pairwise genetic distances fall below a certain value. This was the approach followed for this study.

Identification of the appropriate methods for determining this threshold for ddRADseq data in particular appears to have been little studied to date. Kamvar et al. [16,17] provide some guidance in their papers describing the use of their R package poppr. In the present study, one of the approaches advocated by Kamvar et al. (the cutoff.predictor function) did not yield what appears to be a biologically meaningful threshold (0.001747). Using this threshold, the 81 included samples were collapsed to only 78 multilocus genotypes (here interpreted as genets). This outcome is consistent with the close physical proximity of some of the samples included here. It is also well below what Guilliams and Hasenstab-Lehman [18] recovered using the same function for the close relative, *Eriodictyon altissimum* (0.034700). Instead of relying on the threshold produced by the cutoff.predictor function, we estimated the position of the calculated gap in the number of inferred multilocus genotypes under the farthest neighbor approach using Figure 7 (0.025). These approaches should result in similar thresholds, as occurred in Guilliams and Hasenstab-Lehman [18], so it is unclear why the cutoff.predictor function failed here.

Applying the threshold based on Figure 7, samples within EOs were collapsed to 25 multilocus genotypes. Spatially extensive EOs that support a large number of ramets, such as Orcutt Hill (EO1) and Pine Canyon (EO2+), have a large number of multilocus genotypes. Conversely, spatially restricted EOs that support relatively few ramets have few multilocus genotypes. For example, the VSFB La Salle EO (13) collapsed to one multilocus genotype, which is consistent with the number of ramets and areal extent of this small EO. Exhaustive sampling of all ramets would be required to estimate the total number of multilocus genotypes within a given EO, but the results described here provide important preliminary information that may be useful for conservation planning.

### 3.3. Phylogenomics

In general, the ddRADseq approach was successful in inferring evolutionary relationships in the *Eriodictyon*. Most branches in the tree had the highest possible statistical support (i.e., ML bootstrap value of 100), and only five branches had ML bootstrap values below 70. The *Eriodictyon* clade had the highest possible statistical support, and in general, samples were resolved in clades by taxon.

Phylogenetic relationships were confirmed for taxa historically of uncertain placement. Both *E*. [*Turricula*] *parryi* and *E*. [*Nama*] *lobbii* have been treated recently in *Eriodictyon* [2] due to the findings of Ferguson [1]. In that study, *Eriodictyon* formed a strongly supported clade (parsimony BS = 100), with *E*. [*Turricula*] *parryi* being the sister to all other samples of *Eriodictyon*. Ferguson inferred *E*. [*Nama*] *lobbii* to be sister to *E. californicum*, but with low to moderate statistical support (parsimony BS = 62) and incomplete taxonomic sampling of *Eriodictyon*. While Ferguson’s findings were well-supported in general, reliance upon a single chloroplast locus (ndhF)—which was common at the time—and incomplete sampling allowed some doubt to persist. Here, *E. lobbii* is a recovered sister to a subclade of *Eriodictyon*. *Eriodictyon parryi* is recovered as a sister to the rest of *Eriodictyon*, as determined by Ferguson, but low support indicates other alternative potential phylogenetic placements in *Eriodictyon*. For this reason, treatment of *E. parryi* in *Eriodictyon* rather than *Turricula* seems warranted.

*Nama rothrockii* was sister to *Turricula* + *Eriodictyon* in Ferguson’s analysis, but with somewhat low statistical support (parsimony BS = 58). *Nama rothrockii* is a perennial herb, an uncommon trait in the Namaceae of California, which it shares with *E. lobbii*. For this reason, and given the placement of *E. lobbii* in *Eriodictyon* in Ferguson’s analysis, it was possible that *N. rothrockii* would be recovered in *Eriodictyon* as well. In the analysis presented here, *N. rothrockii* samples form a clade with the highest possible support (ML bootstrap value = 100) that is sister to *Eriodictyon* (inclusive of *E*. [*Turricula*] *parryi*) with strong support (ML bootstrap value = 90). Given the strong phylogenetic placement of *N. rothrockii* as more closely related to *Eriodictyon* than *Nama*, a new name for *N. rothrockii* will be required so that only monophyletic groups are recognized taxonomically (Guilliams and Hasenstab-Lehman, in prep).

The results presented here shed light on the taxonomic circumscriptions and evolutionary history of *E. altissimum*, *E. angustifolium*, *E. californicum*, and *E. capitatum*. Explicitly [9] or implicitly connected by a mosaic of morphological similarity in leaf and flower features, these taxa were of special interest in this study due to the rarity and listing status of *E. altissimum* and *E. capitatum*. Wells [9] speculated that *E. altissimum* may have arisen through hybridization between *E. californicum*, with which it shares inflorescence (open panicle, glabrous axes) and flower (glabrous calyx) features, and *E. capitatum*, with which it shares leaf features (e.g., linear leaves). Here we find maximum statistical support for the present taxonomic circumscriptions of *E. altissimum* and *E. capitatum*, which are reciprocally monophyletic and sister in our analysis. *Eriodictyon californicum* is not closely related to either taxon in our analysis, nor is the linear-leaved *E. angustifolium*.

## 4. Materials and Methods

### 4.1. Sampling

Sampling for Objectives 1 to 3 of this study focused on obtaining high-quality, silica-dried tissues from throughout as much of the range of *E. capitatum* as possible. For *E. capitatum*, 12 samples were included from Orcutt Hills (EO1), 16 samples were included from Pine Canyon (EO2+), 13 samples were included from Hollister Ranch (EO5), 12 samples were included from 35th St. (EO9), 12 samples were included from La Salle (EO13), and 20 samples were included from the Air Field (EO14). Note that EO15 and EO16, which are geographically proximal to EO2, were recently designated. For simplicity, we included them in EO2 (as EO2+) in this study. It was not possible to obtain samples from the Dangermond location (EO12) for this study. For Objective 4, silica-dried tissues were collected from throughout the range of the genus. Outgroup sampling for Objective 4 included *Wigandia* Kunth (one sample), core *Nama* L. (two samples), and *Nama rothrockii* (three samples). A typical tissue collection included 1–2 fresh green leaves placed in a clean, labeled coin envelope. Coin envelopes were aggregated in small batches into small ziplock bags containing silica gel. All tissues have been deposited in the Tissue Bank at the Santa Barbara Botanic Garden. Vouchers for the study have been deposited at the Clifton Smith Herbarium (SBBG) at the Santa Barbara Botanic Garden. Data for each sample included in the study are given in Supplementary Table S1.

### 4.2. DNA Extraction

Dried silica material was ground with a multi-sample Bead Beater tissue homogenizer into a fine powder and extracted using a modified CTAB protocol [19] with the following change: incubation in the CTAB extraction buffer with proteinase K at 65 degrees for 3–4 h, with overnight precipitations. Half the samples went through a final cleaning step with a Zymo DNA Clean and Concentrator-25 kit (Zymo Research, Irvine, CA, USA). Extractions were quantified on a Qubit fluorometer using the Qubit Double Stranded High Sensitivity Assay Kit (Invitrogen, Carlsbad, CA, USA) to check for a suitable genomic DNA quantity. DNA quality was assessed by visualization on an agarose gel following gel electrophoresis.

### 4.3. Library Preparation

Libraries were prepared for high-throughput sequencing using a restriction site-associated DNA sequencing (RADseq) protocol. The RADseq approach is a genomic DNA reduction technique that isolates sequencing regions of genomic DNA near a set of restriction enzyme cut sites. The approach is cost-effective and can be repeated in large numbers of samples to produce a reduced subset of the genome in each individual. After sequencing, the data are re-assembled into loci, anchored by the presence of the restriction enzyme cut site [20,21], and subsequently, single nucleotide polymorphisms (SNPs) are identified across those loci. Double digestion RADseq (ddRADseq) was selected for its ease of use and cost-effective implementation for generating a large SNP dataset from non-model organisms [22,23]. In ddRADseq, two restriction enzymes are used to fragment genomic DNA, followed by size selection of the fragments. This results in sequencing libraries with loci randomly distributed throughout the genome of the study system. This method has been employed in numerous studies and has typically resulted in hundreds to thousands of loci sufficient to address typical population genetics studies in model and non-model organisms.

ddRADseq libraries were prepared in the genetics laboratory at the Santa Barbara Botanic Garden. Library preparation and barcode design follow Tripp et al. [24], with the following modifications: Total genomic DNA was fragmented using the MseI and EcoRI restriction enzymes. A total of 150–500 ng of genomic DNA was added to a reaction solution consisting of: 8.2 µL molecular grade water, 1.15 µL Tango Buffer (Fisher Scientific, Carlsbad, CA, USA), 0.6 µL of 1.0 M NaCl, 0.3 µL (1.0 mg/mL) Bovine Serum Albumin (BSA), 0.28 µL High Fidelity EcoRI (Fisher Scientific), and 0.12 µL MseI (Fisher Scientific). Digestion reactions were incubated at 37 °C for 15 min, followed by an incubation step at 65 °C for 45 min.

Barcodes and adaptors containing an Illumina PCR priming site and the EcoRI cut site were prepared by integrated DNA technologies (Coralville, IA, USA) and follow the design of Tripp et al. [24]. Each ligation reaction consisted of the entire double restriction digestion reaction containing the fragmented genomic DNA to which we added 1.0 µL of 1.0 µM EcoRI adaptor + barcodes, 0.072 µL water, 0.1 µL 10× T4 buffer, 0.05 µL of 1.0 M NaCl, 1.0 mg/mL BSA, 1.0 10 nM MseI adaptor, and 0.165 µL T4 DNA ligase. Reactions were mixed, centrifuged, and incubated for 16 h at 16 °C, then heat-inactivated at 65 °C for 10 min. These restriction-ligation reactions were diluted 1:10 using 0.1× TE buffer.

We ran two separate 20 µL PCR reactions per restriction-ligation product [22]. PCR reactions contained: 8.6 µL molecular grade water, 4.0 µL Phusion High Fidelity Buffer (New England Biolabs, Ipswich, MA, USA), 0.5 µL of 10 µM Illumina primer 1 (IDT; (A*A*TGATACGGCGACCACCGAGATCTACACTCTTTCCCTACACGACGCT CTTCCGATCT), 0.5 µL of 10 µM Illumina primer 2 (IDT; C*A*AGCAGAAGACGGCATACGA GCTCTTCCGATCTGTAAG), 1.6 µL of 2.5 mM dNTPs, 0.1 µL Phusion High Fidelity DNA polymerase (New England Biolabs, Ipswich, MA, USA), and 5 µL diluted restriction-ligation reaction. Each PCR reaction used the following cycling parameters: 98 °C for 60 s; 25 cycles of 98 °C for 20 s; 60 °C for 30 s; 72 °C for 40 s; 72 °C for 10 m; 4 °C hold. Gel electrophoresis and imaging were used as a qualitative assay to ensure PCR amplification of fragments at the desired 300–400 bp range for each sample. Successful PCR amplifications were cleaned with Zymo DNA Clean and Concentrator kits (Zymo Research, Irvine, CA, USA), then pooled.

### 4.4. Size Selection, Library Quantification, and Sequencing

The genomic library was sent to the University of California Riverside (UCR) Institute for Integrative Genome Biology Core Instrumentation Facility for size selection. Libraries were size-selected on a 1.5% agarose gel cassette for fragments between 350 and 550 bp in length. The libraries were quality checked with a Bioanalyzer 2100 (Agilent, Santa Clara, CA, USA) at UCR to ensure library quality and concentration prior to sequencing each pooled library on a NextSeq 500 (Illumina, La Jolla, CA, USA), each as a single lane of 1 × 75 single end base pair (bp) reads under the rapid run setting at UCR. 

### 4.5. Data Processing, SNP Calling

Raw sequence reads were demultiplexed by UCR using custom scripts. Read pools were cleaned and quality checked using FastQC [25]. To assemble loci and generate phylip files for downstream phylogenomic analyses, cleaned sequence data were further processed with ipyrad v. 0.9.69 [26,27] on an iMac Pro with 10 cores.

Sequence assembly was performed using the de novo assembly in ipyrad, using the following parameters: ddrad datatype, phred quality score minimum of 33, with the parameters clustering threshold at 0.85, mindepth of 6 and maximum barcode mismatch of 0, 35 bps minimum length of sequences after the adaptor trim, a maximum of 2 alleles per site in consensus sequences, 0.02 max_Ns_consens, 0.05 heterozygotes, 0.2 SNPs per locus, and 8 max_indels.

We generated two different datasets in ipyrad to address different components of the study. Dataset 1 included only samples of *E. capitatum* and was filtered to include loci with a single SNP per locus. This dataset was used in analyses associated with Objectives 1 to 3. In Dataset 2, sampling was expanded to include nearly all other *Eriodictyon* taxa, along with outgroups in the Namaceae. This dataset was used to accomplish Objective 4 of the study.

### 4.6. Population Genomic Analyses

Population genomic summary statistics were calculated by element occurrence in the program Stacks v. 2.60 [28,29] from the VCF output from ipyrad for Dataset 1. Each EO was treated as a population for the purposes of running these analyses. Settings used to calculate the population genetics statistics included: --min-populations = 2, indicating the minimum number of populations a locus must be present in to process a locus; -R, --min-samples-overall = 50 for the minimum percentage of individuals across the dataset required to process a locus; -H was applied to prune unshared SNPS to reduce haplotype-wise missing data; --write-random-snps restricts data analysis to one random SNP per locus. Statistics include the number of individuals from each population/EO (N), the mean individuals genotyped at each locus (n), the number of private alleles (Private), the mean frequency of the major allele (P), observed heterozygosity (Ho), expected heterozygosity (He), nucleotide diversity (Pi), and the mean Wright’s inbreeding coefficient (*F*_IS_).

Genetic differentiation between pairwise combinations of *E. capitatum* EOs was examined using *F*_ST_. *F*_ST_ is a common measure of genetic differentiation, with higher values indicating a greater degree of genetic differentiation between populations and lower values indicating a greater number of shared alleles. These values were calculated under the Stacks pipeline using the “populations” program.

Multi-variate statistical methods were used to examine patterns in the genetic dataset. These methods are largely exploratory in nature and do not have strong assumptions about an underlying genetic model, such as the presence of Hardy-Weinberg equilibrium or the absence of linkage disequilibrium [30]. A principal coordinates analysis (PCoA) was performed on the genlight matrix in the R package dartR [31]. PCoA is a statistical procedure that transforms a large number of variables into fewer composite variables, or PCs. These composite variables can be used to identify possible structures or clusters of genotypes within and among populations of individuals in the dataset.

To further assess the population structure of *E. capitatum*, two different analyses were performed. First, a phylogenetic analysis was performed on Dataset 1 using maximum likelihood (ML) in the program RAxML [32]. The analysis was performed on the CIPRES Science Gateway v3.3 [33]. Statistical confidence was assessed using ML bootstrapping. Second, Bayesian clustering was implemented in the program STRUCTURE [34] with ipyrad v. 0.9.77 analysis tools [35] in a Jupyter notebook [36] on the VCF output from populations and converted to hdf5 file format. STRUCTURE identifies genetic subdivisions in the data and then assigns samples to these subdivisions using an admixture model, assuming correlation of allele frequencies without prior knowledge of sample locality, for subdivisions (K) = 1–10, with *n* = 10 for each K value. STRUCTURE was run using an imap dictionary to color individuals; minmap = 0.5, which filters SNPs to only include those that have data for a 50% proportion of samples in every group; and mincov = 50 for the entire dataset. We set the MCMC chain to a burn-in of 20,000, followed by 100,000 MCMC iterations. To obtain the most likely value of K, the LnP(K) and deltaK were evaluated under the Evanno method [37]. Results from the separate 10 MCMC analyses were summarized in a barplot, with each genetic cluster assigned a different color. Each sample is colored by the estimated proportion of genotypes shared with each cluster.

### 4.7. Clonality

Clonality in *E. capitatium* was assessed using Dataset 1. For this, the multilocus genotypes within the samples were inferred using the R package poppr v2 [16,17]. Multilocus genotypes are unique combinations of alleles across at least two loci [16]. There are several ways to construct multilocus genotypes from a dataset. Naïve string matching is one approach that collapses samples together only when they are identical. This approach is not appropriate for calling multilocus genotypes using high-throughput sequencing, however, as samples may vary slightly owing to hypervariable loci and common artifacts of high-throughput sequencing such as missing data [17].

The resulting VCF file was read into R and converted to a genlight object. Individuals with low read counts (<5000) were removed from the dataset prior to converting the genlight object to a snpclone object for use in the R package poppr. The threshold of genetic similarity below which samples were collapsed into multilocus genotypes was determined in two ways. First, a threshold was calculated using the cutoff.predictor function in poppr. The cutoff.predictor function identifies the largest gap between inferred numbers of multilocus genotypes for all thresholds and can be run under nearest neighbor, UPGMA, and farthest neighbor clustering methods. The farthest neighbor method was used as it is the most conservative [17]. In addition, the initial largest gap between the inferred numbers of multilocus genotypes for all thresholds was also identified using a plot-based approach. The mlg.filter function was used to assign multilocus genotypes.

### 4.8. Phylogenomics

Phylogenetic analyses were performed using maximum likelihood (ML) in the program RAxML [32] on the CIPRES Science Gateway v3.3 [33]. Statistical confidence was assessed using ML bootstrapping, with bootstrapping halted automatically by the program. Analyses were performed with the RAxML HPC2 on the XSEDE tool using default parameters. The resulting trees were visualized using the program FigTree v1.4.3.

## 5. Conclusions and Recommendations

*Eriodictyon capitatum* is a narrowly distributed shrub endemic to western Santa Barbara County (Santa Barbara, CA, USA), where it is known from only 10 EOs. Here, high-throughput DNA sequence data were analyzed to investigate genetic diversity within and among all accessible EOs; to determine the extent of genetic isolation among EOs; to examine clonality within EOs; and to examine the taxonomic circumscriptions of *E. capitatum*, *E. altissimum*, *E. angustifolium*, and *E. californicum* through phylogenomic analysis. Population genetic analyses of *E. capitatum* revealed a pattern of strong genetic differentiation by location/EO. The clonality assessment showed that certain small EOs may support relatively few multilocus genotypes. The phylogenomic analyses strongly supported the present-day taxonomic circumscriptions of both *E. altissimum* and *E. capitatum*, showing them to be reciprocally monophyletic and sister with strong support. Taken together, these results paint a picture of an evolutionarily and morphologically distinct species known from relatively few, genetically isolated stations.

The results of this study were used to develop a list of conservation recommendations. Most broadly, both *E. capitatum* and *E. altissimum* were strongly supported as monophyletic in the phylogenomic analysis and should continue to be managed as evolutionarily distinct rare plant taxa under applicable federal, state, and local laws. Similarly, *E. capitatum* EOs were found to be genetically differentiated using population genetic and phylogenetic approaches. The preservation of each EO should be prioritized to conserve the overall genetic diversity of *E. capitatum*. Care should be taken to avoid the unintended movement of genetic material (e.g., pollen, seeds) between EOs. Conversely, because plants of *E. capitatum* are largely self-incompatible [14], it may be desirable to develop an ex situ research program to explore the feasibility of using hand-pollination crosses between genets, sourced from the same and/or different EOs, to bolster seed production. If such a study in the greenhouse resulted in increased seed production, then a potential in situ program might be designed in collaboration with government agency personnel and land managers.

The results presented here are consistent with general patterns of plant biodiversity in California as they pertain to high levels of endemism in coastal Central California. The California Floristic Province (CA-FP) has long been recognized as a global biodiversity hotspot, owing to the region’s large proportion of endemic taxa and high degree of habitat loss [38,39]. In a state-wide analysis of endemism, Stebbins and Major [40] suggested CA-FP subdivisions of roughly similar size and tabulated their properties. The range of *E. capitatum* lies near the border of their Central Coast and Southern California subdivisions, both of which were reported to harbor the highest levels of endemism in the state under multiple of their metrics. A recent, spatially explicit study based on georeferenced herbarium specimens [41] estimated that species-rank endemism in the CA-FP is approximately 36.9% (1846 endemic species of 5006 total native species), and endemism of minimum-rank taxa (inclusive of subspecies and varieties) was even higher, at 42.5% (2612 endemic minimum-rank taxa of 6143 native minimum-rank taxa). The Central Western California Region (CW), where *E. capitatum* occurs, contains a large number of CA-FP endemic species (740) and the greatest number of endemic species when scaled to unit area (20 endemic species per 1000 km^2^) [41]. Using a different spatially explicit approach, Baldwin and colleagues [42] showed high species richness for portions of this same region, along with concentrations of grid cells with high values of weighted endemism. Although the results of the present study pertain to only two of the many endemic plant species of California’s Central Coast, insights into the history of these two plants support regional findings and may be more broadly representative of overall patterns of biodiversity in the state.

## Figures and Tables

**Table 1:** Summary population genomic statistics for each element occurrence (EO) averaged across polymorphic loci. Statistics include the number of individuals from each population (*N*), the mean individuals genotyped at each locus (*n*), the number of private alleles (Private), the mean frequency of the major allele (P), observed heterozygosity (*H*o), expected heterozygosity (*H*e), nucleotide diversity (Pi), and the mean Wright’s inbreeding coefficient (*F*_IS_).

EO	*N*	*n*	Private	P	*H*o	*H*e	Pi	*F*_IS_
Orcutt Hill—EO1	12	7.819	31,142	0.917	0.059	0.118	0.127	0.186
Pine Canyon—EO2+	16	10.325	32,246	0.919	0.056	0.116	0.123	0.205
Hollister Ranch—EO5	13	6.961	21,888	0.936	0.057	0.087	0.095	0.093
35th St.—EO9	12	9.641	15,293	0.942	0.089	0.071	0.078	−0.009
La Salle—EO13	12	8.875	8572	0.967	0.046	0.041	0.045	0.001
Air Field—EO14	20	15.675	14,296	0.977	0.034	0.029	0.031	−0.004

**Table 2:** *F*_ST_ values for each pair of element occurrences (EOs).

	EO2+	EO5	EO9	EO13	EO14
EO1	0.114	0.170	0.187	0.214	0.226
EO2+		0.163	0.178	0.168	0.215
EO5			0.266	0.319	0.326
EO9				0.354	0.357
EO13					0.417

**Table 3:** Summary statistics associated with the STRUCTURE analysis.

K	Nreps	lnPK	lnPPK	deltaK	estLnProbMean	estLnProbStdev
1	10	0.000	0.000	0.000	−5.277 × 10^5^	2.517 × 10^3^
2	10	8.039 × 10^4^	2.439 × 10^4^	5.419	−4.473 × 10^5^	4.502 × 10^3^
3	10	5.600 × 10^4^	1.607 × 10^4^	4.925	−3.913 × 10^5^	3.264 × 10^3^
4	10	3.993 × 10^4^	8.172 × 10^2^	0.193	−3.514 × 10^5^	4.224 × 10^3^
5	10	3.911 × 10^4^	1.418 × 10^4^	1.601	−3.123 × 10^5^	8.859 × 10^3^
6	10	2.493 × 10^4^	1.243 × 10^4^	8.368	−2.873 × 10^5^	1.485 × 10^3^
7	10	1.250 × 10^4^	1.154 × 10^6^	557.149	−2.748 × 10^5^	2.072 × 10^3^
8	10	−1.142 × 10^6^	1.993 × 10^6^	1.248	−1.417 × 10^6^	1.597 × 10^6^
9	10	−3.135 × 10^6^	3.679 × 10^6^	5.829	−4.552 × 10^6^	6.311 × 10^5^
10	10	5.434 × 10^5^	0.000	0.000	−4.009 × 10^6^	2.164 × 10^6^

**Table 4:** Multilocus genotypes inferred by element occurrence (EO).

EO	# Samples	# Multilocus Genotypes
Orcutt Hill—EO1	11	9
Pine Canyon—EO2+	16	9
Hollister Ranch—EO5	13	3
VSFB 35th St.—EO9	11	2
VSFB La Salle—EO13	12	1
VSFB Airfield—EO14	18	1

## Data Availability

All sequence data generated for this project have been uploaded to the NCBI Sequence Read Archive (https://www.ncbi.nlm.nih.gov/sra (accessed on 31 October 2023)). Sample accession numbers can be found in Supplementary Table S1.

## Supplementary Materials

The following supporting information can be downloaded at: https://www.mdpi.com/article/10.3390/plants13010090/s1, Supplementary Table S1: Sample information; Supplementary Table S2: Inferred multilocus genotypes.
